# Machine Learning Prediction of Discharge Destination in Patients with Parkinson’s Disease; A Nationwide Cohort Study

**DOI:** 10.21203/rs.3.rs-8399223/v1

**Published:** 2026-01-08

**Authors:** Hikaru Kamo, Tejas R Mehta, Matthew Remz, Rachael M. Burke, Anne Brooks, Adrianne Smiley, Michael S. Okun, Christopher W. Hess

**Affiliations:** Fixel Institute for Neurological Diseases; Fixel Institute for Neurological Diseases; Fixel Institute for Neurological Diseases; Fixel Institute for Neurological Diseases; Parkinson’s Foundation; Parkinson’s Foundation; Fixel Institute for Neurological Diseases; Fixel Institute for Neurological Diseases

**Keywords:** Parkinson’s Disease, hospitalization, machine learning, discharge disposition

## Abstract

**Background.:**

Non-home discharge after hospitalization is common among patients with Parkinson’s disease (PD) and is associated with adverse outcomes. Early identification of patients likely to require post-acute facility care may improve discharge planning.

**Methods.:**

We conducted a retrospective cohort study using a nationwide healthcare database, including adults aged ≥50 years hospitalized with PD between November 2017 and June 2023. The first hospitalization of each patient was defined as the index admission. Discharge destination was categorized as home, facility, or in-hospital death. Data were split into training (80%) and testing (20%) sets. Random forest models were developed to predict discharge destination, and performance was evaluated using area under the receiver operating characteristic curve (AUC). An elastic net logistic regression model was additionally developed for facility discharge.

**Results.:**

Among 281,664 index hospitalizations, 48.0% were discharged home, 44.8% to a facility, and 7.2% died in-hospital. In the test set, random forest models achieved AUCs of 0.775 for home discharge, 0.774 for facility discharge, and 0.832 for in-hospital death. An elastic net model achieved an AUC of 0.752, and a seven-item risk score (fracture history, dementia, transfer admission, fall history, marital status, insurance type, hospital region) identified a high-risk group with a 73.8% facility discharge rate, compared with 40.6% in the low-risk group.

**Conclusions.:**

Using nationwide claims data, this disease-specific prediction model identified discharge destination in hospitalized patients with PD. A simplified, interpretable risk score enables early risk stratification at admission and may facilitate multidisciplinary discharge planning and post-acute care allocation.

## Introduction

Parkinson’s disease (PD) is a progressive neurodegenerative disorder characterized by both motor symptoms and a broad range of non-motor impairments, including cognitive decline, dysphagia, and falls.^1–3^ As the disease advances, increasing functional dependency and medical complications often limit patients’ ability to return home after hospitalization, resulting in discharge to rehabilitation facilities, long-term care institutions, or nursing homes.^4,5^ Institutional discharge is associated with worse outcomes, including greater functional decline, higher readmission and mortality rates, and increased healthcare utilization, making discharge destination a clinically important outcome in PD.^6,7^ Discharge destination in PD reflects a complex interaction of patient characteristics and hospitalization-related factors, such as age, comorbidity burden, dysphagia, aspiration pneumonia, and falls.^8–10^ Early identification of patients at risk for institutional discharge may enable targeted discharge planning, multidisciplinary rehabilitation, and timely community-based support, with the potential to promote home discharge and reduce subsequent readmissions. Machine learning (ML) approaches have been increasingly used to predict discharge destination in general medical and surgical populations, demonstrating moderate to good discrimination, with reported area-under-the-curve (AUC) values ranging from approximately 0.7 to 0.9 using routinely collected structured data.^11–17^ However, to date, no ML-based prediction model has been developed specifically for patients hospitalized with PD. Using a large national administrative claims database, we developed and internally validated ML models to predict discharge destination as home, facility, or in-hospital death among patients hospitalized with PD and examined patient- and hospital-level factors associated with these outcomes.

## Methods

### Study Design and Data Source

Hospitalization records were obtained from a large, nationwide administrative claims database (PINC AI Healthcare Database, Premier Inc., Charlotte, NC, USA).^18^

**Figure 1:**
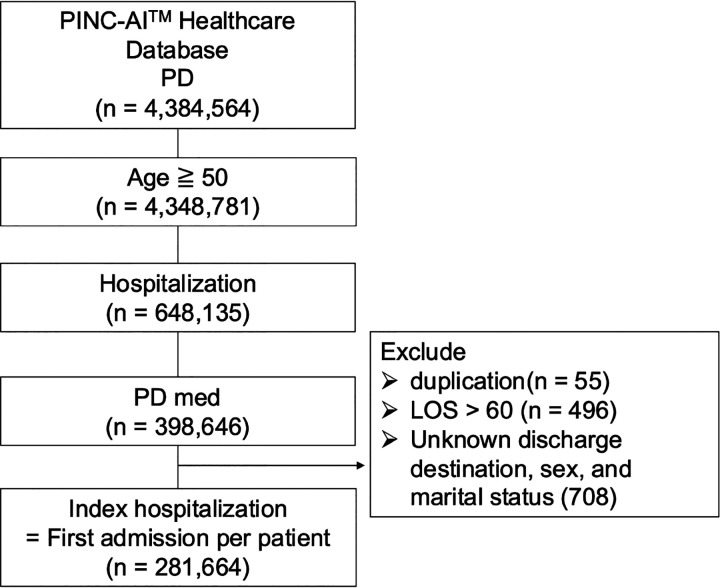
Study flow diagram Hospitalized patients with Parkinson’s disease (ICD-10-CM code G20) aged 50 years or older were identified from the PINC AI Healthcare Database (Premier Inc., Charlotte, NC, USA). After excluding admissions longer than 60 days, duplicate records, and cases with missing demographic information, 281,664 patients were included in the final cohort. Discharge destinations were classified as home (48.0%), facility (44.8%), or in-hospital death (7.2%).

Eligible patients were adults aged 50 years or older with a diagnosis of PD (ICD-10-CM code G20) who were hospitalized between November 13, 2017, and June 30, 2023. For patients with multiple hospitalizations, only the first hospitalization during the observation period was defined as the index admission and included in the analysis. Patients were excluded if their index hospitalization exceeded 60 days, if duplicate records were identified, or if demographic information such as sex, discharge disposition, or marital status was missing or coded as unknown. After applying these criteria, a total of 281,664 patients were included in the final analytic cohort (Figure 1). The primary outcome was discharge destination, categorized as home discharge, facility discharge, or in-hospital death. Home discharge referred to patients returning to their own residence or another nonmedical residential setting. Facility discharge included transfers to rehabilitation hospitals, skilled nursing facilities, long-term care institutions, or other medical or custodial facilities. In-hospital death was defined as death occurring during the index hospitalization or following transition to inpatient hospice care. In the final cohort, 48.0% were discharged home, 44.8% to a post-acute facility, and 7.2% died during hospitalization.

### Covariates

To identify factors associated with discharge destination, both patient-level and hospital-level variables were evaluated. Patient-level characteristics included age, sex, race, the source of admission, marital status (married, unmarried, or other), residence type (urban or rural), and insurance type (private, Medicare, or Medicaid). Clinical factors comprised dementia, dysphagia, aspiration pneumonia, fracture, Charlson Comorbidity Index score, length of stay, and rehabilitation frequency. Hospital-level variables included teaching status, bed capacity, and geographic region (Northeast, Midwest, South, or West). The source of admission was categorized as home, transfer, or other, following standard UB-04 point-of-origin definitions. Admissions from non–health care settings, including private residences and nonmedical residential facilities, were classified as home. Transfers from another health care facility or program were classified as transfer, whereas legal, disaster-related, and unspecified origins were grouped as other.

### Machine Learning–Based Prediction of Discharge

To identify factors associated with discharge destination (home, facility, or in-hospital death) among hospitalized patients with PD, a series of predictive analyses was performed using both patient- and hospital-level variables. The dataset was randomly split into training (80%) and testing (20%) sets. Records with missing or indeterminate demographic or clinical data were excluded. All analyses were performed using R (version 4.4.0).

Discharge outcomes were modeled using a random forest algorithm, incorporating demographic, clinical, and hospital-level attributes. Model parameters were optimized through cross-validation, and internal validation was performed with three-fold cross-validation.

Predictive performance was evaluated using the area under the receiver operating characteristic curve (AUC) with 95 % confidence intervals, as well as sensitivity, specificity, positive and negative predictive values, overall accuracy, the F1-score, and confusion matrices based on the optimal Youden threshold. Ninety-five % confidence intervals for AUC values were computed using 2,000 bootstrap resamples, although point estimates are presented in the Results section for clarity. Model calibration was assessed through calibration plots comparing predicted and observed probabilities, and variable importance was determined from the mean decrease in Gini impurity. Because the in-hospital mortality rate was low (7.2%), a class-weight adjustment was applied in the mortality model to address class imbalance, whereas the home and facility discharge models were trained without weighting.

For the primary outcome of facility discharge, an additional analysis using an Elastic Net logistic regression model was performed to enhance interpretability and allow risk stratification. This model combined L1 and L2 regularization for variable selection and coefficient shrinkage. Standardized coefficients were used to construct a Facility Discharge Risk Score scaled from 0 to 70 points, and patients were categorized into low-, medium-, and high-risk strata. Observed facility discharge rates were compared across strata to evaluate discrimination and clinical utility.

### Ethical Considerations and Reporting

This study used de-identified administrative data and therefore did not require institutional review board approval or informed consent, in accordance with regulations governing research using anonymized datasets. The PINC AI^™^ Healthcare Database is a proprietary dataset available under license from Premier, Inc. All analytic scripts are available from the corresponding author upon reasonable request. This study followed the STROBE and RECORD reporting guidelines. All analytic scripts used for data preprocessing, model training, and evaluation were written in R and are available from the corresponding author upon reasonable request.

## Results

### Patient Characteristics

Among 281,664 hospitalizations for patients with PD, discharge destinations were home in 48.0%, facility in 44.8%, and in-hospital death in 7.2%. Table 1 summarizes patient and hospital characteristics stratified by discharge outcome. Patients discharged to a facility were, on average, older (77.7 ± 8.1 years) than those discharged home (75.6 ± 8.9 years) and had a higher comorbidity burden (Charlson Comorbidity Index: 2.2 ± 1.9 vs. 2.0 ± 2.0). The prevalence of dementia (42.6%), dysphagia (14.9%), and aspiration pneumonia (7.7%) was substantially higher among facility discharges compared with home discharges (27.6%, 9.2%, and 5.2%, respectively). Patients who died in-hospital had the highest rates of dementia (53.7%), aspiration pneumonia (27.8%), and PEG tube placement (1.1%). Hospital-level factors also varied by outcome: patients discharged to facilities were more often treated in large hospitals (>500 beds) and teaching institutions, and more frequently admitted from other healthcare facilities (17.9% vs. 9.0% for home discharges). Length of stay was longest among facility discharges (median 5 days [IQR 3–8]) and shortest among home discharges (median 3 days [IQR 2–5]). Rehabilitation sessions were most frequent in the facility group (7.3 ± 7.3), indicating higher intensity of inpatient therapy.

### Predictive Model for Home Discharge

In the full inpatient cohort, the tuned Random Forest model achieved an AUC of 0.775 for predicting discharge to home. Calibration analysis demonstrated agreement between predicted probabilities and observed home discharge rates (Figure 1). Using the optimal Youden threshold of 0.483, the model achieved a sensitivity of 0.658 and a specificity of 0.736. The positive predictive value was 0.695 and the negative predictive value was 0.702, with an overall accuracy of 0.699 and an F1-score of 0.676. The corresponding confusion matrix consisted of 17,716 true positives, 7,778 false positives, 21,642 true negatives, and 9,197 false negatives. Key contributors to model performance included length of stay, rehabilitation frequency, age, dementia, fracture history, and the source of admission, indicating that both clinical status and preadmission context strongly influenced discharge outcomes. Variable importance scores for all predictors are presented in Supplementary Table S1.

### Prediction model for Facility Discharge

**Figure 2:**
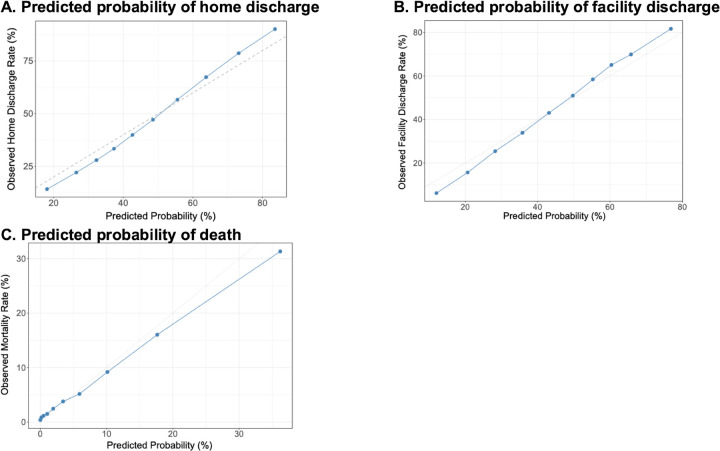
Calibration curve for discharge prediction using the tuned Random Forest model. **A.** Calibration plot of the Random Forest model predicting home discharge (AUC = 0.775). **B.** Random Forest model predicting discharge to post-acute or long-term care facilities (AUC = 0.774). **C.** Weighted Random Forest model predicting in-hospital mortality (AUC = 0.832). All models demonstrated good discrimination and close agreement between predicted and observed probabilities across risk deciles. AUC, area under the curve; RF, random forest.

The random forest model showed good accuracy for predicting discharge to post-acute or long-term care facilities (AUC = 0.774; Figure 2B). Using the optimal Youden threshold of 0.423, the model yielded a sensitivity of 0.77 and a specificity of 0.62. The positive predictive value was 0.624 and the negative predictive value was 0.767. Overall accuracy was 0.687, with an F1-score of 0.689. The confusion matrix included 19,533 true positives, 11,779 false positives, 19,188 true negatives, and 5,833 false negatives. Predicted probabilities were well calibrated against observed outcomes. Longer length of stay and higher rehabilitation frequency were the strongest predictors of facility discharge. Other important factors included dementia, fracture history, transfer admission, and overall comorbidity burden (Supplementary Table S1). Patients with higher medical complexity or greater functional dependence were more likely to be discharged to a facility.

**Figure 3:**
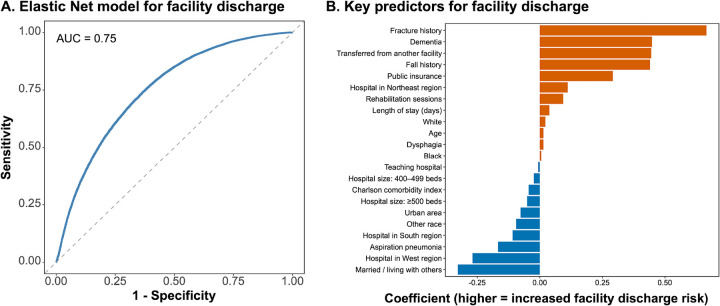
Predictive performance and key predictors for facility discharge using an Elastic Net model **A.** Receiver operating characteristic (ROC) curve showing the predictive performance of the Elastic Net model for facility discharge (AUC = 0.75). The model demonstrated good discrimination for identifying patients at risk of institutional discharge. **B.** Variable importance plot showing key predictors associated with facility discharge in the Elastic Net model. Positive coefficients indicate higher risk of discharge to a facility, whereas negative coefficients indicate higher likelihood of home discharge.

To improve interpretability, an Elastic Net logistic regression model was developed using the same dataset (Figures 3A and 3B). The model demonstrated moderate discrimination, achieving an AUC of 0.752. Using the optimal Youden threshold of 0.397, the model yielded a sensitivity of 0.739 and a specificity of 0.635, with an overall accuracy of 0.682 and an F1-score of 0.677. The confusion matrix included 18,754 true positives, 11,304 false positives, 19,663 true negatives, and 6,612 false negatives. This model identified seven independent predictors: fracture history, dementia, transfer admission, fall history, marital status, insurance type, and hospital region (Table 2). Facility discharge was more frequent in patients with fracture, dementia, or transfer admission, whereas being married or living with others and admission to western-region hospitals were associated with home discharge. Based on these coefficients, a Facility Discharge Risk Score was created, allowing each predictor to contribute proportionally to the total risk. The score classified patients into low-, medium-, and high-risk groups, which corresponded to observed facility discharge rates of 40.6%, 64.0%, and 73.8%, respectively. This seven-item scoring system provides a straightforward and clinically meaningful way to anticipate discharge needs and support early planning for post-acute care.

### Prediction model for Death

A Random Forest model with class-weight adjustment was developed to predict in-hospital mortality among all admitted patients. The model incorporated 21 demographic, clinical, and hospital-level variables, including admission source, comorbidities, and treatment-related factors. Using 80% of the dataset for training and 20% for testing, the model achieved an AUC of 0.832, demonstrating strong discrimination. Using the optimal Youden threshold of 0.079, the model achieved a sensitivity of 0.781 and a specificity of 0.745. Although the positive predictive value was modest (0.192) because of the low prevalence of in-hospital mortality, the negative predictive value remained high at 0.978, indicating reliable exclusion of mortality risk.

Overall accuracy was 0.748 with an F1-score of 0.308, reflecting the inherent imbalance of the outcome. The calibration analysis demonstrated close agreement between predicted and observed mortality probabilities across deciles of risk (Figure 2C), suggesting appropriate model calibration. The top contributing predictors of mortality included age, CCI, length of stay, aspiration pneumonia, and dysphagia, followed by dementia, PEG tube, and urinary catheter (Supplementary Table S1). These findings highlight the predominant influence of age, comorbidity burden, and markers of clinical severity or feeding difficulty on mortality risk among hospitalized patients with PD.

## Discussion

In this nationwide cohort study using a large administrative claims database, we developed and internally validated ML models to predict discharge destination among patients hospitalized with PD. The models demonstrated good discrimination and highlighted clinically interpretable factors associated with facility discharge and in-hospital death. These findings support the feasibility of claims-based risk stratification to inform discharge planning in PD.

### Machine learning discharge prediction in PD

Previous studies have applied ML algorithms to predict discharge disposition or inpatient outcomes across various medical and surgical populations, such as patients with hip fracture, spinal cord injury, traumatic brain injury, and total joint arthroplasty.^16,17,19–24^ These studies demonstrated that ML approaches outperformed traditional comorbidity indices in predicting mortality, complications, and nonhome discharge, with AUC values typically ranging from 0.7 to 0.9. However, these models were designed for general populations and did not account for the disease-specific characteristics of PD. In PD, progressive cognitive deterioration, hallucinations, emotional and behavioral disturbances, dysphagia, aspiration pneumonia, recurrent falls, fractures, and frailty frequently coexist, together exerting profound effects on both physical function and social independence.^25,26^ These factors not only reflect disease severity during hospitalization but also impact the ability to adapt to post-discharge living environments and can add to the factors determining the level of recommended long-term care. Moreover, the fluctuating course of symptoms, difficulty in prognostication, and wide spectrum of non-motor manifestations make discharge planning in PD inherently complex, creating a multifaceted and longitudinal decision-making process that differs fundamentally from that of other medical conditions.^27^ Patients with frailty or swallowing impairment are particularly vulnerable to in-hospital death or institutional discharge. Insufficient access to palliative care and family support especially at advanced stages are factors potentially preventing a discharge to a home setting.^8,28–30^

In this study, we developed and validated a discharge prediction model specifically for patients with PD using a large nationwide claims database. Both the random forest and Elastic Net models demonstrated good discriminatory performance, identifying dementia, dysphagia, fracture, transfer admission, and comorbidity burden as major predictors of discharge to post-acute or long-term care facilities. These factors collectively reflect the physical and cognitive vulnerability characteristics of advanced PD, highlighting structural risks that are may not be fully captured by conventional models developed for general medical populations. Dementia limits decision-making capacity and independence in ADL, often making home discharge unrealistic, whereas dysphagia and aspiration pneumonia substantially increase nutritional and caregiving demands, reducing the feasibility of home care. Fracture and fall history indicate physical frailty and delayed recovery after hospitalization, frequently necessitating continuous assistance or supervised living environments. Transfer admissions from other facilities generally represent patients who already require complex medical or nursing support, reflecting a low likelihood of returning home even at the time of admission. In contrast, being married and admission to hospitals in western regions were associated with a higher likelihood of home discharge, suggesting that social and regional factors such as family support and the availability of community-based care strongly influence discharge outcomes. Furthermore, the simplified risk score derived from the selected predictors provided a practical tool for early identification of patients at high risk for nonhome discharge, enabling individualized discharge planning and multidisciplinary intervention.

### Clinical and policy implications

The findings of this study have important implications for both clinical practice and healthcare policy. The predictive model and simplified risk score developed in this study provide a practical framework for early stratification of PD inpatients at high risk for institutional discharge, enabling timely and individualized discharge planning. Previous studies in general medical populations have shown that early identification of discharge risk using ML facilitates timely multidisciplinary interventions and reduces readmissions.^31–34^ In PD, however, complex vulnerabilities including cognitive decline, falls, fractures, dysphagia, and frailty have a profound influence on functional independence and discharge outcomes, yet disease-specific prediction models remain scarce.^25,26^

In the present study, the Elastic Net model identified fracture, dementia, and fall history as strong predictors of facility discharge, suggesting that modifiable risk factors related to physical and cognitive decline play a central role in discharge outcomes. Early preventive strategies such as fall-prevention programs, cognitive screening, and osteoporosis management may possibly improve home discharge potential and reduce long-term care dependence. Transfer admission, although not a modifiable factor, reflects patients who already require complex medical or nursing support and thus should prompt early initiation of coordinated post-acute and discharge planning by multidisciplinary teams. In contrast, being married or living with others and was associated with higher home discharge rates, suggesting that family support and well-developed community care systems could possibly facilitate return to home. The association between public insurance (Medicare or Medicaid) and facility discharge likely reflects socioeconomic vulnerability and disparities in access to home- and community-based services, underscoring the importance of early social work involvement and linkage to regional care networks.

The seven-item Facility Discharge Risk Score derived from these predictors offers a practical bedside tool for identifying high-risk patients at admission and for guiding early interventions such as rehabilitation, swallowing therapy, caregiver education, and coordination with community-based resources. Moreover, regional differences in discharge disposition highlight the uneven distribution of healthcare resources and post-acute care capacity, emphasizing the need for equitable policy approaches to long-term care delivery.^35–38^ Integrating such data-driven discharge prediction into clinical workflows may improve home discharge rates, reduce institutionalization and healthcare costs, and ultimately promote sustainable, patient-centered long-term care for individuals with PD.

### Limitations

This study has several limitations. First, the analysis relied on administrative claims data, which are subject to coding errors and misclassification, and key clinical variables such as PD severity, functional status, cognition assessments, and social determinants of health were not directly available. Second, discharge destination based on claims may not fully capture baseline residence or caregiving context, which could introduce classification bias. Third, although we used a large national database and performed internal validation, the models were not externally validated; performance may differ across health systems and patient populations. Finally, as an observational study using claims-derived variables, residual confounding and unmeasured factors may influence associations between predictors and outcomes.

## Conclusions

Using a large nationwide claims database, this study developed and validated the first disease-specific prediction model for discharge destination among hospitalized patients with PD. The Elastic Net model identified key clinical and social determinants of facility discharge, including fracture, dementia, fall history, marital status, insurance type, and hospital region, and demonstrated good discriminatory performance. The simplified seven-item risk score provided an interpretable and practical tool for identifying high-risk patients at admission and for guiding early, multidisciplinary discharge planning. Integration of such data-driven prediction models into clinical workflows may improve home discharge rates, optimize post-acute care resource allocation, and promote sustainable, patient-centered care for individuals with PD.

## Supplementary Files

This is a list of supplementary files associated with this preprint. Click to download.

- SupplementaryTableS1.docx
- GraphicalAbstract.png

## Figures and Tables

**Table 1.** Baseline characteristics of hospitalized patients with Parkinson’s disease by discharge destination

Variable	Category	Home	Facility	Death
Age (years)		75.6 – 8.9	77.7 – 8.1	79.3 – 7.9
Sex	F	53740 (39.7%)	53171 (42.1%)	7416 (36.7%)
	M	81501 (60.3%)	73043 (57.9%)	12793 (63.3%)
Race	Asian	3256 (2.4%)	2302 (1.8%)	538 (2.7%)
	Black	9012 (6.7%)	9352 (7.4%)	1506 (7.5%)
	Other	7609 (5.6%)	5750 (4.6%)	1066 (5.3%)
	White	115364 (85.3%)	108810 (86.2%)	17099 (84.6%)
Marital status	Alone	64518 (47.7%)	72764 (57.7%)	11020 (54.5%)
	With others	70723 (52.3%)	53450 (42.3%)	9189 (45.5%)
Residence type	RURAL	15392 (11.4%)	15079 (11.9%)	2220 (11%)
	URBAN	119849 (88.6%)	111135 (88.1%)	17989 (89%)
Insurance type	Private	13126 (9.7%)	6739 (5.3%)	1171 (5.8%)
	Public	122115 (90.3%)	119475 (94.7%)	19038 (94.2%)
Geographic region	MIDWEST	28631 (21.2%)	31173 (24.7%)	4466 (22.1%)
	NORTHEAST	23594 (17.4%)	24758 (19.6%)	3511 (17.4%)
	SOUTH	63147 (46.7%)	55318 (43.8%)	9503 (47%)
	WEST	19869 (14.7%)	14965 (11.9%)	2729 (13.5%)
Teaching hospital	NO	69015 (51%)	64015 (50.7%)	10160 (50.3%)
	YES	66226 (49%)	62199 (49.3%)	10049 (49.7%)
Bed capacity	<100	9617 (7.1%)	8817 (7%)	1098 (5.4%)
	100–199	20773 (15.4%)	19608 (15.5%)	2894 (14.3%)
	200–299	23951 (17.7%)	23481 (18.6%)	3704 (18.3%)
	300–399	22695 (16.8%)	21816 (17.3%)	3518 (17.4%)
	400–499	15926 (11.8%)	14645 (11.6%)	2435 (12%)
	>500	42279 (31.3%)	37847 (30%)	6560 (32.5%)
Dementia	NO	97854 (72.4%)	72433 (57.4%)	9366 (46.3%)
	YES	37387 (27.6%)	53781 (42.6%)	10843 (53.7%)
Dysphagia	NO	122785 (90.8%)	107439 (85.1%)	14932 (73.9%)
	YES	12456 (9.2%)	18775 (14.9%)	5277 (26.1%)
Aspiration pneumonia	NO	128269 (94.8%)	116451 (92.3%)	14589 (72.2%)
	YES	6972 (5.2%)	9763 (7.7%)	5620 (27.8%)
Fracture	NO	128247 (94.8%)	102993 (81.6%)	18458 (91.3%)
	YES	6994 (5.2%)	23221 (18.4%)	1751 (8.7%)
Fall history	NO	123821 (91.6%)	98349 (77.9%)	17945 (88.8%)
	YES	11420 (8.4%)	27865 (22.1%)	2264 (11.2%)
Polypharmacy	NO	132912 (98.3%)	124152 (98.4%)	19986 (98.9%)
	YES	2329 (1.7%)	2062 (1.6%)	223 (1.1%)
Urinary catheter	NO	132743 (98.2%)	122245 (96.9%)	19525 (96.6%)
	YES	2498 (1.8%)	3969 (3.1%)	684 (3.4%)
PEG tube	NO	134824 (99.7%)	125486 (99.4%)	19982 (98.9%)
	YES	417 (0.3%)	728 (0.6%)	227 (1.1%)
Admission source	clinic	11886 (8.8%)	7676 (6.1%)	807 (4%)
	home	109914 (81.3%)	94861 (75.2%)	14526 (71.9%)
	other	1221 (0.9%)	1097 (0.9%)	232 (1.1%)
	transfer	12220 (9%)	22580 (17.9%)	4644 (23%)
Charlson Comorbidity Index		2.0 – 2.0	2.2 – 1.9	2.9 – 2.2
Length of stay (days)		3 [2–5]	5 [3–8]	5 [2–10]
Rehabilitation frequency		3.8 – 5.4	7.3 – 7.3	2.8 – 5.6

Values are presented as mean ± standard deviation, median [interquartile range], or n (%), as appropriate. Discharge destinations were categorized into home, facility, and in-hospital death. Facility discharge included transfers to skilled nursing facilities, inpatient rehabilitation units, or long-term care institutions.

**Table 2.** Facility Discharge Risk Scoring System and Risk Stratification Derived from the Elastic Net Mode

Predictor	Coefficient (β)	Standardized score (0–10 scale)	Direction of association
Fracture history	0.666	10	↑
Dementia	0.448	6.7	↑
Transferred from facility	0.445	6.7	↑
Fall history	0.44	6.6	↑
Public insurance	0.292	4.4	↑
Married / living with others	−0.328	−4.9	↓
Hospital in West region	−0.269	−4.0	↓
Risk stratification based on total score			
Risk tier	Total score range	Observed facility discharge (%)	Mean total score
Low	≤ 25	40.6	9.6
Medium	26–45	64	33.9
High	≥ 46	73.8	52.9

The seven most influential predictors were standardized to a 10-point scale (maximum total = 70 points). Patients were classified into three risk categories (Low, Medium, High) according to total score, showing a stepwise increase in facility discharge rates from 40.6% to 73.8%.

## Data Availability

Data used in this study were obtained from a proprietary administrative claims database and are not publicly available. Access to the data may be granted by the data vendor to qualified researchers for purposes of replicating procedures and results, subject to data use agreements and applicable regulations. The analytic code used in this study is available from the corresponding author upon reasonable request.
